# A systematic review of the application and utility of geographical information systems for exploring disease-disease relationships in paediatric global health research: the case of anaemia and malaria

**DOI:** 10.1186/1476-072X-12-1

**Published:** 2013-01-10

**Authors:** Ashley Mariko Aimone, Nandita Perumal, Donald C Cole

**Affiliations:** 1 Division of Epidemiology, Dalla Lana School of Public Health, 155 College Street Health Science Bld, 6th floor, Toronto, ON, M5T 3M7, Canada; 2 Child Health Evaluative Sciences, The Hospital for Sick Children, 525 University Ave, Toronto, ON, M5G 1X8, Canada; 3 Division of Global Health, Dalla Lana School of Public Health, 155 College Street Health Science Bld, Suite 400, Toronto, ON, M5T 3M7, Canada

**Keywords:** Geographic information systems, Infants and preschool children, Malaria, Anemia, Rural health, Developing countries, Systematic review, Adapted review guidelines

## Abstract

Malaria and anaemia are important health problems among children globally. Iron deficiency anaemia may offer protection against malaria infection and iron supplementation may increase the risk of malaria-related hospitalization and mortality. The nature and mechanism of these relationships, however, remain largely unresolved, resulting in concern and uncertainty around policies for non-selective iron supplementation in malaria endemic areas. Use of geographical information systems (GIS) to investigate this disease-disease interaction could contribute important new information for developing safe and effective anaemia and malaria interventions. To assess the current state of knowledge we conducted a systematic review of peer-reviewed and grey literature. Our primary objective was to qualitatively assess the application and utility of geographical concepts or spatial analyses in paediatric global health research. The secondary objective was to identify geographical factors that may be associated with anaemia and malaria prevalence or incidence among children 0–5 years of age living in low- and middle-income countries. Evaluation tools for assessing the quality of geographical data could not be found in the peer-reviewed or grey literature, and thus adapted versions of the STROBE (Strengthening The Reporting of Observational Studies in Epidemiology) and GRADE (Grades of Recommendation, Assessment, Development and Evaluation) methods were used to create reporting, and overall evidence quality scoring systems. Among the 20 included studies, we found that both malaria and anaemia were more prevalent in rural communities compared to urban areas. Geographical factors associated with malaria prevalence included regional transmission stability, and proximity to a mosquito breeding area. The prevalence of anaemia tended to vary inversely with greater or poorer access to community services such as piped water. Techniques for investigating geographic relationships ranged from simple descriptive mapping of spatial distribution patterns, to more complex statistical models that incorporated environmental factors such as seasonal temperature and rain fall. Including GIS in paediatric global health research may be an effective approach to explore relationships between childhood diseases and contribute key evidence for safe implementation of anaemia control programs in malaria endemic areas. Further, GIS presentation of ecological health data could provide an efficient means of translating this knowledge to lay audiences.

## Review

### Background

#### Pediatric public health problems in a global context

Childhood malnutrition and infection make up a large proportion of the global health disease burden [1]. Anaemia and malaria, in particular, are among the most important public health issues worldwide, especially in low and middle-income countries [2,3]. There are several potential causes of anaemia, with iron deficiency as the largest contributor in approximately half of all cases [2]. Generally, anaemia is a marker of both poor health and poor nutrition, and severe anaemia has been shown to increase the risk of child mortality [4,5]. Other important causal factors of anaemia include parasitic infections, such as malaria [6]. Most malaria infections are caused by *Plasmodium falciparum*, and is a leading cause of morbidity and mortality among children in sub-Saharan Africa [7,8]. Malaria causes anaemia primarily through haemolysis, the destruction of red blood cells [9]. Along with other haeme-related disorders, such as inherited thalassaemia and haemoglobinopathies, it has also been suggested that iron deficiency may offer protection against malaria infection [10]. On the contrary, evidence from a large randomized controlled trial in Pemba, Zanzibar (> 30,000 participants) suggested that providing iron supplements to young children living in a malaria endemic area who are iron replete may increase the risk of malaria-related hospitalization and mortality [11]. This trial was the first of its kind to be adequately powdered to investigate mortality as a primary outcome; and the findings have fuelled much debate within the scientific community over the nature of the interaction between malaria and anaemia, and the potential biological mechanisms that drive this relationship. As a result, concern and uncertainty were generated within the global health community, especially around policies for routine and non-selective iron supplementation in areas where malaria is highly prevalent.

#### Geographic information systems in health research

A geographical information system (GIS) constitutes a system of hardware and software used for storage, management, retrieval, manipulation, analysis, modeling, and mapping of geographical data [12]. It is an “enabling technology” that is relatively easy to use and access by non-experts in geography or cartography with basic computer literacy and map reading skills. There are several potential advantages of using GIS to investigate global health issues [13]: 1) it allows the exploration of the role of geographical or environmental factors in the prevalence (or incidence) of a health outcome of interest; 2) the combination of cartography and multivariate analysis allows investigation of complex spatial relationships (e.g. linking people and health outcomes to space and time); 3) GIS software enables the presentation of research findings in a visual manner that can be easily interpreted across disciplines; and 4) the technique can be applied to a range of analysis units, which may provide insight into relationships between health outcomes and other social, demographic, or economic variables at various jurisdictional levels. Although the use of GIS technology is expanding on a global scale (as indicated by ESRI licensing records alone), potential limitations of its use may include the lack of available geographically referenced data and the prohibitive cost of collecting such information, especially in remote or low-resource settings. More recently, however, the development of open-access GIS platforms, as well as initiatives by the United Nations (UN) and other government organizations (e.g. IDRC, WHO and CDC) have increased the accessibility and availability GIS software for low- and middle-income countries [14].

Geospatial analysis can facilitate identification of the most vulnerable populations in terms of a disease outcome of interest, and thus contribute valuable information for planning targeted public health interventions. Assessments of the relationships between anaemia and malaria have not generally included geographical factors. Considering that the burden of anaemia and malaria is highest among children from poorer parts of low- and middle-income countries, these two prominent health problems could share common ecological and geographical settings. A systematic exploration and appraisal of current evidence regarding the application of GIS and spatial analysis in the investigation of these problems could be beneficial.

#### Objectives of the systematic review

Our primary objective was to determine how geographical concepts or spatial analyses have been applied to the investigation of anaemia and malaria outcomes among young children in low-resource settings; as well as discuss the potential utility of these applications. The secondary objective was to identify geographical factors that may be associated with anaemia and malaria prevalence or incidence among children 0–5 years of age living in low- and middle-income countries

## Methods

### Peer-reviewed literature search

Relevant peer-reviewed journal databases (PUBMED, MEDLINE, EMBASE, SCOPUS, WEB OF SCIENCE) were searched using the following terms and operators: (spatial OR “space-time” OR geostat* OR gis OR “geographic* information system*”) AND (child* OR infant* OR newborn OR neonat*) AND (“low income” OR “middle income” OR “developing country” OR “low resource”) AND (anemia OR anaemia OR “iron deficiency” OR hemoglobin OR haemoglobin OR malaria). The titles and abstracts of all records were screened by two reviewers using the following relevance criteria:

• Population: study population includes children ≤ 5 years of age living in low or middle income countries (World Bank classification)

• Approach: use of geographical or ecological-level data with/without spatial analysis

• Co-approach: complimentary or comparative use of individual-level data (or other “non-geographical” factors) and corresponding analysis methods

• Outcomes: anaemia, malaria

• Exclusions: animal studies, in-vitro studies

### Grey literature search and hand searches

Grey literature sources were sought by hand searching the reference lists of selected studies; searching publication lists of major international organizations involved in global pediatric health research and promotion (e.g. WHO and UNICEF); and conducting online searches for companies, institutions, or research groups involved in GIS work with a public health focus (e.g. Infonaut, Sault Ste. Marie Innovation Centre, MEASURE DHS, MARA, and MAP).

### Data Screening and extraction

The full text versions of records from peer-reviewed and grey literature sources that met relevance criteria, based on the title and abstract, were retrieved and the data extracted onto pre-designed forms. For those records with titles and abstracts that did not contain enough information to assess relevance, the full texts were retrieved and screened. In order to assess inter-reviewer reliability, a subset of 30 records was randomly selected and the titles/abstracts independently screened by the two reviewers using the relevance criteria described above. The results of these independent screenings were compared through the calculation of a Kappa statistic, which quantifies the measure of agreement between independent reviewer assessments, taking into account the probability of chance agreements [15]. When there was disagreement in terms of relevance, the two reviewers met and reached consensus. If consensus could not be reached, a third independent reviewer was consulted to resolve the discrepancy.

### Quality assessment

Literature quality was not included as a relevance criterion, and thus quality assessments were conducted after the full text screening and selection processes. As it was anticipated that the majority of the health outcome data reviewed would be observational in nature, the quality of reporting these outcomes were evaluated using the STROBE method (Strengthening The Reporting of Observational Studies in Epidemiology) [16,17]. An example of a similar evaluation tool for assessing the quality of reporting geographical data could not be found in the peer-reviewed or grey literature; however, the ‘Metadata Standards’ of the US National Centre for Geographic Information and Analysis (NCGIA) guidelines were adopted as evaluation criteria. Under the ‘Metadata’ model, transparency in documentation is a requirement for meeting this standard, and thus studies that included a description of how the spatial data were produced were given a higher quality rating than those that did not. These criteria were included in an adapted version of the STROBE checklist (Table 1), which was used to create a binomial scoring system (e.g. 1 = criterion met, 0 = criterion not met). Final score percentages were based on the number of reporting quality criteria met divided by the number of criteria that were relevant (e.g. criterion for case control studies will not apply to evaluation reports).

**Table 1:** Adapted STROBE Statement—checklist of items that should be included in reports of observational studies (including additions/adaptations for accommodating geographical data)

	**Item No**	**Recommendation**
**Title and abstract**	1	(*a*) Indicate the study’s design with a commonly used term in the title or the abstract
		(*b*) Provide in the abstract an informative and balanced summary of what was done and what was found
**Introduction**		
Background/rationale	2	Explain the scientific background and rationale for the investigation being reported
Objectives	3	State specific objectives, including any prespecified hypotheses
**Methods**		
Study design	4	Present key elements of study design early in the paper
Setting	5	Describe the setting, locations, and relevant dates, including periods of recruitment, exposure, follow-up, and data collection
Participants	6	(*a*) *Cohort study*—Give the eligibility criteria, and the sources and methods of selection of participants. Describe methods of follow-up
		*Case*–*control study*—Give the eligibility criteria, and the sources and methods of case ascertainment and control selection. Give the rationale for the choice of cases and controls
		*Cross*-*sectional study*—Give the eligibility criteria, and the sources and methods of selection of participants
		(*b*) *Cohort study*—For matched studies, give matching criteria and number of exposed and unexposed
		*Case*–*control study*—For matched studies, give matching criteria and the number of controls per case
Variables	7	Clearly define all geographic variables, outcomes, exposures, predictors, potential confounders, and effect modifiers. Give diagnostic criteria, if applicable
Data sources/ measurement	8*	For each geographic and outcome variable of interest, give sources of data and details of methods of assessment (measurement). Describe comparability of assessment methods if there is more than one group
Bias	9	Describe any efforts to address potential sources of bias
Study size	10	Explain how the study size was arrived at
Quantitative variables	11	Explain how quantitative geographic and outcome variables were handled in the analyses, including how geographic variables were handled in the creation of attribute tables, thematic maps, etc. using GIS software (as well as the name and version number of the software used). If applicable, describe which groupings were chosen and why.
Statistical methods	12	(*a*) Describe all statistical methods, including spatial analyses and those used to control for confounding.
		(*b*) Describe any methods used to examine geographic and outcome subgroups and interactions
		(*c*) Explain how missing geographic and outcome data were addressed
		(*d*) *Cohort study*—If applicable, explain how loss to follow-up was addressed
		*Case*–*control study*—If applicable, explain how matching of cases and controls was addressed
		*Cross*-*sectional study*—If applicable, describe analytical methods taking account of sampling strategy
		(*e*) Describe any sensitivity analyses
**Results**		
Participants	13*	(a) Report numbers of individuals at each stage of study—e.g. numbers potentially eligible, examined for eligibility, confirmed eligible, included in the study, completing follow-up, and analysed
		(b) Give reasons for non-participation at each stage
		(c) Consider use of a flow diagram
Descriptive data	14*	(a) Give characteristics of study participants (e.g. demographic, clinical, social) and information on exposures and potential confounders. Summarize geographic characteristics of study area (if applicable).
		(b) Indicate number of participants with missing data for each geographic and outcome variable of interest
		(c) *Cohort study*—Summarise follow-up time (e.g., average and total amount)
Outcome data	15*	*Cohort study*—Report numbers of outcome events or summary measures over time
		*Case*–*control study*—Report numbers in each exposure category, or summary measures of exposure
		*Cross*-*sectional study*—Report numbers of outcome events or summary measures
Main results	16	(*a*) Give unadjusted estimates and, if applicable, confounder-adjusted estimates and their precision (e.g., 95% confidence interval). Make clear which confounders were adjusted for and why they were included
		(*b*) Report category boundaries when geographic or continuous outcome variables were categorized
		(*c*) If relevant, consider translating estimates of relative risk into absolute risk for a meaningful time period
Other analyses	17	Report other analyses done—e.g. spatial analyses, analyses of subgroups and interactions, and sensitivity analyses
**Discussion**		
Key results	18	Summarise key results with reference to study objectives
Limitations	19	Discuss limitations of the study, taking into account sources of potential bias or imprecision. Discuss both direction and magnitude of any potential bias
Interpretation	20	Give a cautious overall interpretation of results considering objectives, limitations, multiplicity of analyses, results from similar studies, and other relevant evidence
Generalisability	21	Discuss the generalisability (external validity) of the study results
**Other information**		
Funding	22	Give the source of funding and the role of the funders for the present study and, if applicable, for the original study on which the present article is based

*Give information separately for cases and controls in case–control studies and, if applicable, for exposed and unexposed groups in cohort and cross-sectional studies.

The overall quality of evidence from selected studies was assessed using the GRADE approach (Grades of Recommendation, Assessment, Development and Evaluation Working Group 2004). Under this approach, assessing the quality of a body of evidence involves consideration of within-study risk of bias, and data quality. The approach specifies four levels of quality: high (+2), moderate (+1), low (0), and very low (≤ −1). Quality ratings may be downgraded or upgraded based on the presence of eight factors [18]: 1) Limitations in design and implementation; 2) Indirectness of evidence; 3) Unexplained heterogeneity or inconsistency of results; 4) Imprecision of results; 5) High probability of publication bias; 6) Large magnitude of effect; 7) All plausible confounding would reduce a demonstrated effect or suggest a spurious effect when results show no effect; 8) Dose–response gradient.

The GRADE approach was originally developed to assess the quality of medical evidence from clinical studies, and there did not appear to be a similar validated evaluation tool for assessing the quality of geographic evidence. There is, however, a sufficiently large body of literature on spatial data quality that could be used to create an adapted version of the GRADE approach. As such, relevant data quality elements extracted and compiled from various sources [19,20], including: 1) Accuracy (a component of quality that can be defined in terms of the entity-attribute-value model); 2) Resolution (the amount of detail that can be discerned in space, time or theme); 3) Consistency (the absence of apparent contradictions in a database); 4) Completeness (lack of errors of omission in a database relative to the database specification).

Verifying the accuracy and completeness of spatial data, while especially important to consider in quantitative analyses and interpretations, was not deemed necessary for addressing the objectives of this qualitative review. However, the resolution (precision) and consistency of geographical maps and mapping techniques used by authors to display and interpret health outcome data was assessed using an adapted GRADE approach. In the case of precision, studies that presented spatial data with insufficient detail to allow appropriate discernment of spatial, temporal, or thematic resolution were given lower quality ratings. In terms of consistency, if there were apparent contradictions in the spatial, temporal, or thematic evidence that were not adequately explained by the investigators, quality ratings were decreased.

### Data synthesis

Due to the observational and heterogeneous nature of the literature retrieved, a qualitative synthesis (rather than a meta-analytic assessment) of the data was performed. Patterns in the data within and between studies were analysed inductively. Methods used to apply geographical concepts or spatial analyses were similarly assessed.

## Results

### Literature search, data synthesis and quality assessment

**Figure 1:**
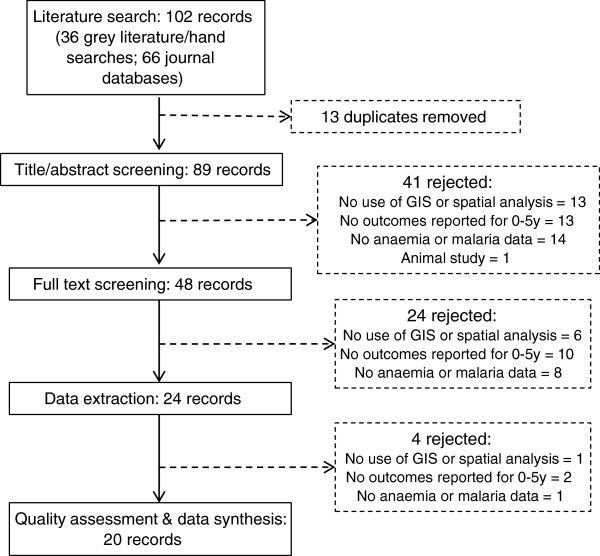
Study flow from literature review to data synthesis.

A total of 102 records were retrieved from peer-reviewed and grey literature sources, as well as through individual hand searches. Of these records, 13 were duplicates and 69 did not meet relevance criteria (Figure 1). The primary reasons for rejection were: 1) lack of data pertaining to a target population in the specified age range of 0–5 years (25 records); and 2) lack of data on anaemia or malaria outcomes (25 records). The Kappa score for the sub-sample of independently reviewed titles/abstracts was 0.61, reflecting a good agreement between the two reviewer assessments [15]. Twenty records remained for inclusion in this review and underwent quality assessments and data synthesis.

A description of all selected studies, ranked according to their reporting quality ratings, is presented in Table 2. Six out of twenty records included anaemia as a primary outcome [2,21-24], while the rest were focused on malaria. The target population in 75% (15/20) of the selected studies and reports were from single or multiple countries in Africa. Three reports, all published by the WHO, pertained to all children globally, and 1 record included a target population from Asia (Indonesia). Reporting quality scores ranged from 41.4% to 88.3%, with the majority of records (13/20) having final scores between 60 and 80%. In terms of the quality of evidence, 4 studies were upgraded once, mainly due to reporting dose–response gradient-like relationships between a geographic factor and the prevalence/incidence of malaria or anaemia outcomes [24-27]. Three studies were upgraded twice due to dose–response relationships and additional control or consideration of confounders and potential biases [21,28,29]. All downgraded records (8/20) were given ratings of −1 due to the indirectness of results (e.g. spatial analysis was applied to whole population data rather those specific to young children; or geographic relationships were inferred from inter-site or cross-country comparisons). 

**Table 2:** Description of selected studies

** Author (year)**	** Source**	**Objectives**	**Target population**	**Target outcome(s)**	**Reporting quality (% score)**	**Quality of evidence (rating)**
Snow (1999b) [30]	Bull World Hlth Org	Estimate age-structured rates of the fatal, morbid and disabling sequelae following expoure to malaria infection under different epiemiolgical conditions.	African population	Malaria	86.67	0
MARA/ARMA (1998) [31]	MARA website	Provide a continental perspective of where, how much, when, why, and who is affected by malaria, and establish a continental database of the spatial distribution of malaria in Africa	children < 10y (excluding infants) in Africa	Malaria	85.00	0
Snow (1998a) [32]	Trans Roy Soc Trop Med Hyg	Develop climate-based model of transmission intensity and estimate annual morbidity and mortality burden of malaria among children in Kenya.	Children 0-10y in Kenya	Malaria	83.87	−1
Schellenberg et al., (1998) [26]	Int Epi Assoc	Study the geographicla pattern of hospital admissions for severe malaria and stability of this pattern over time in Kilifi Distric, Kenya.	Children < 5y in Kenya	Malaria	81.25	1
Giardina et al. (2012) [28]	PloS One	Provide spatially explicit burden estimates of malaria using survey data and Bayesian geostatistical zero-inflated binomial models.	Children 6–59 months in Senegal	Malaria	78.13	2
WHO (2010) [33]	WHO website	Document success in reducing global malaria burden by summarizing information received from 160 malaria-endemic countries/areas and updating analyses presented in previous annual report.	All population groups with malaria data reported to WHO.	Malaria	77.59	−1
Root (1999) [34]	Int J Pop Geog	Map and describe distribution of under-five mortality at provincial level and examine degree to which socio-economic factors and regional disease environments are responsible for spatial patterns.	Children < 5y in 20 sub-Saharan African countries	Malaria	74.19	−1
Snow (1999a) [35]	Parasitology Today	Define spatial limits of populations exposed to risk of malaria infection in Africa and obtain best estimate of malaria attributable mortality among infants and children.	Children 0-4y in Africa	Malaria	72.41	−1
WHO (2008b) [7]	WHO website	Rreview progress in controlling malaria burden, implementing national policies and strategies on malaria control, funding to support malaria control, and evidence generation on the epidemiological impact of malaria control programmes.	All population groups with malaria data reported to WHO.	Malaria	70.69	−1
Hightower et al., (1998) [27]	Am J Trop Med Hyg	Illustrate usefulness of Differential Geographical Positioning System (DGPS) maps to produce a highly accurate base map in a tropical area.	Children < 5 years in Siaya district, Western Kenya	Malaria	69.35	1
Mbogo (1995) [36]	Am J Trop Med Hyg	Evaluate the transmission of *P*. *falciparum*by vector populations relative to the incidence of severe malaria infections.	Children 0-4y from nine sites in Kenya	Malaria	62.50	−1
Mbogo (1993) [25]	Am J Trop Med Hyg	Examine dynamics of *P*. *falciparum* transmission by vector populations in relation to the incidenc of severe malaria infections.	Children 1-4y from two study sites in Kilifi District, Kenya	Malaria	61.29	1
Anthony et al., (1992) [37]	Am J Trop Med Hyg	Report findings of a 15-month malaria investigation and identify factors contributing to its origin, exacerbation and persistence.	Children 0-4y in remote highland community of Oksibil, Indonesia	Malaria	59.86	0
Gordon (2004) [38]	WHO website	Describe environmental factors that affect child health (including parasitic infections such as malaria).	Children < 5y worldwide	Malaria	41.38	0
WHO (2008a) WHO [2]	WHO website	Collect and present information on anaemia prevalence by country and WHO region.	All population groups [Children 0.5-4.99y, 5–14.99y, (non) pregnant women, men, elderly]	Anaemia	88.33	−1
Magalhaes (2011) [24]	PLoS Medicine	Estimate the geographical risk profile of anaemia while accounting for malaria, malnutrition, and helminth infections. Estimate the risk of anaemia attributable to these factors, and the number of anaemia cases in preschool-aged children for 2011.	Children 1-4y in Burkina Faso, Ghana, and Mali	Anaemia	87.50	1
Greenwell (2006) [29]	Population Association America	Examine the utility of using child hemoglobin measures (collected in population-based studies) as an indicator for monitoring malaria morbidity.	Children 6–59 months in five sub-Saharan African countries	Anaemia / Malaria	84.38	2
Mainardi (2012) [21]	Int J Geo Info Sci	Re-assess spatial heterogeneity and anisotropy of moderate and severe anaemia using variograms and geographically weighted regression (GWR) models.	Children < 5y in 173 regions of 20 sub-saharan African countries.	Anaemia	76.67	2
Snow (1994) [22]	Acta Topica	Describe and quantify clinical burden of malaria in communities with markedly different levels of *P*. *falciparum* transmission in East Africa.	Children 0-9y in Kilifi, Kenya and Ifakara, Tanzania	Anaemia / Malaria	70.97	−1
Tanzanian NBS and ICF International (2012) [39]	MEASURE DHS website	Summarize findings of the 2010 Tanzanian DHS, and provide an atlas of maps intended to easily communicate regional differences in maternal and child health.	Women (15-49y) and children (6-59 m) in Tanzania	Anaemia	66.67	0

### GIS applications and spatial analyses of malaria and anaemia among children

Table 3 summarizes the results from selected records in terms of GIS applications and the observed geographic factors associated with malaria or anaemia among young children. The use of GIS and spatial analysis ranged from simple descriptive maps of study areas [22,25,36], to more complex depictions of environmental or spatial predictors of health outcomes. The most common GIS application was describing the distribution and spatial patterns of anaemia and malaria-related outcomes (e.g. disease prevalence or incidence, vector transmission or parasitology) using thematic mapping, such as choropleth or dot density [2,7,24,27,29,31,33,34,37-39]. The Mapping Malaria Risk in Africa (MARA) project, and more recent initiative, Malaria Atlas Project (MAP), have reported extensive use of GIS combined with fuzzy logic (MARA) and posterior predictive modeling (MAP), to estimate malaria risk according to levels of parasite endemicity, transmission stability, and climate suitability [26,30,32,35,40]. GIS has also been combined with other types of geostatistical methods – such as Bayesian geostatistical models [24,28], and geographically weighted regression [21] – to estimate the spatial distribution of burden and environmental predictors of malaria and anaemia (e.g. climate or urbanization).

**Table 3:** Summery of GIS applications and geographic factors associated with malaria and anaemia among children 0-5y of age living in low- or middle-income countries

** Author (year)**	**GIS application**	**Geographic factors of malaria or anaemia**
	**Malaria**	
Anthony et al., (1992) [37]	Dot map of malaria incidence in one of the study villages.	Malaria point prevalence varied within and across villages in Indonesia. Incidence of malaria infections greatest in Yapimakot (39.1%), followed by Dabolding (34.95), Kabiding (31.9%) and Kutdol (28.6%). Prevalence of malaria ~50% lower among populations living in areas of forest-covered mountain slopes above the valley compared to villagers.
Giardina et al. (2012) Giardina et al. [28]	Geospatial analysis, remote sensing data, and choropleth maps used to estimate environmental/climatic predictors of malaria.	Prevalence of malaria varied across survey locations in Senegal (lowest in northern regions, highest in the sourthern regions). High geographical variation in parasitaemia prevalence, including urban (1.3%) vs. rural (8.47%) differences (reduced odds for urban areas by 81%, 95% BCI: 55%-93%).
Gordon (2004) [38]	Choropleth and symbology maps used to depict worldwide prevalence estimates and other related geographic features such as climate suitability for vector transmission.	Annual deaths from malaria in 2002 by WHO region highest in Africa (978,661) and lowest in Europe (44).
Hightower et al., (1998) [27]	GIS used to perform spatial analyses and link location information to parasitology and entomology databases.	Prevalence of parasitemia tended to decrease with increasing household distance from larval habitat (p = 0.3437) except during the dry month of September. Average number of trapped *An*. *gambiae* mosquitoes was related to the distance of the household to the nearest breeding site for September (p = 0.0039), but not wet month of June (p = 0.1530). Opposite relationship was found for *An*. *funestus*(June p = 0.0191, September p = 0.6608).
MARA/ARMA (1998) MARA/ARMA [31]	Various thematic maps used to depict relevant environmental (e.g. climatic) and population characteristics (e.g. density), and disease prevalence/incidence data.	Childhood (0-4y) population exposed to malaria mortality risk was higher in areas with 50% malaria transmission stability than areas with 90%. In Kenya the number of children < 5y who die or develop clinical malaria varies across areas of high, medium, low, or unstable malaria endemicity. In Mali an inverse U-shaped association found between malaria prevalence and distance to a water source (total population estimate).
Mbogo (1993) [25]	Vector map of study area.	Prevalence of asymptomatic infections (with or without parasitaemia concentration ≥ 5000/uL) was higher in rural area of Sokoke compared to Kalifi town, Kenya. Higher proportion of children recruited from Sokoke reported to the District Hopsital with febrile illness and high parasitemia.
Mbogo (1995) [36]	Vector map of study area.	Spatial patterns of severe disease varied across study sites indpendently of transmission intensity and entomological innoculation rate (EIR).
Root (1999) Root [34]	Choropleth maps to depict spatial patterns of <5 mortality in 20 sub-Saharan African countries.	High mortality rates in East/South Africa and in vicinity of Lake Victoria represented heterogeneity in disease environments, indicating spatial impact and correlation between intensity of malaria transmission and observed mortality patterns.
Schellenberg et al., (1998) [26]	Choropleth maps used to depict quintiles of severe malaria presenting to District Hospital and layout of all-weather roads.	Admission rates significantly higher in children living within 5 km from hospital (31.6/1000 child-years at risk) compared to those > 25 km away (5.0 per 1000 child-years at risk). Children living > 2.5 km away from nearest road were significantly less likely to be admitted compared to those living < 0.5 km (Adj RR = 0.47, 95%CI: 0.3-0.9).
Snow (1998a) [32]	Dot density map of projected population distribution according to modelled predictions of regions of stable malaria endemicity.	High transmission intensity conditions identified around Lake Victoria (affecting 677,000 children < 5y). Largest number of children 0-4y exposed to areas of moderate stable malaria endemicity. Highest risk of malaria mortality and hospital admission in areas of high and moderate stable malaria endemicity, respectively.
Snow (1999a) [35][30]	Dot density map of population distribution from communities exposed to at least 50% probability of malaria transmission according to a fuzzy logic climate model.	Wide geographical variation in estimates of malaria mortality in childhood. Deaths in hospital due to malaria per 1000 catchment childhood population highest in Sukutu, The Gambia (range 0.33-2.8) compared to other sites.
Snow (1999b) [30][30]	Thematic maps of climate suitability for stable transmission, interpolated population density, and zones of malaria risk in Africa.	Higher median mortality and morbidity rates in areas of stable transmission with ≥ 0.2 climate suitability than malaria risk area in South Africa with ≥ 0.5 climate suitability.
WHO (2008b) [7]	Choropleth maps of global incidence of malaria (and malaria related deaths) in 2006.	Variation in estimated burden of malaria (cases and deaths) in 2006 among children < 5y within and across 30 high burden countries.
WHO (2010) [33]	Choropleth maps of geographical distribution of confirmed malaria cases/1000 population.	Variation in estimated malaria cases among children < 5y across 24 selected countries between 2000–2009.
	**Anaemia**	
Mainardi ( 2012) [21]	Spatial distribution of anaemia prevalence, including comparison between countries, and association with urabanizaiton.	Geographical variation in average proporiton of children with moderate or severe anaemia. Localization/urbanization was inversely associated with moderate and severe anaemia (OLS). Increased median time to a water source was significantly associated with lower prevalence of moderate (p < 0.01), but not severe anaemia (GWR). Widespread anaemia prevalence observed in mainly inland regions in West Africa, and a few specific areas in Eastern and central Africa.
Snow 1994 [22]	Vector map of study areas in Kenya and Tanzania.	Higher prevalence of parasitaemia among children 0-4y in Ifakara compared to Kilifi. Higher prevalence of severe anaemia among children 0-4y in Kilifi than Ifakara.
WHO (2008a) [2]	Choropleth maps of global anaemia prevalence and public health significance by country.	Prevalence of anaemia among pre-school aged children (0.5-4.99y) highest in Africa (global range 23.1 to 67.6%).
Tanzanian NBS and ICF International 2012 [39]	Choropleth maps of anaemia prevalence by region.	Anaemia prevalence ranged from 42% among two in-land regions (Rukwa and Kilimanjaro) to 78% in the northern island region of Unguja.
Greenwell 2006 [29]	Choropleth maps of anaemia or malaria prevalence, as well as malaria transmision by country (vector) or overall (raster). Overlayed dot density maps were used to show cluster locations.	Children in areas of moderate malaria prevalence were at highest risk of severe anaemia. The validity of haemoglobin measurements was dependent on whether the assessment was conducted during a high malaria transmission season.
Magalhaes 2011 [24]	Dot density map of anaemia prevalence by DHS location. Choropleth maps of predictive geogrpahical risk or variation of anaemia or Hb concentration.	Mean haemoglobin was lowest in Burkina Faso, and a large spatial cluster of low mean haemoglobin and high anaemia risk was predicted for an area shared by Burkina Faso and Mali.

According to the WHO reports included in this review (Table 3), annual childhood mortality from malaria in 2002 were highest in Africa (978, 661 deaths/year) , followed by South-East Asia (57, 877 deaths/year) [38], and this global pattern has been consistently supported by malaria incidence data from 160 endemic countries up to 2009 [7,33], as well as global malaria risk tables and prevalence distribution maps produced by the Malaria Atlas Project [40]. Variations in malaria prevalence within countries have also been reported in both Asia and Africa. Anthony et al. found lower prevalence in areas of Indonesia among populations living outdoors on forest-covered mountain slopes compared to villagers living in houses [37]. In Senegal, Giardina’s group reported higher malaria prevalence in rural areas compared to urban areas [28], while in Kenya, a higher prevalence of parasitaemia was found among young children from households that were closer to mosquito larval habitats, particularly during months with heavier rainfall [27]. Similarly, Schellenberg et al. reported higher malaria-related hospital admission rates among children who lived closer to a hospital (< 5 km vs. > 25 km) and the nearest road (< 0.5 km vs. > 2.5 km) [26]. Spatial variations in malaria mortality and morbidity rates, clinical malaria incidence, and severe malaria infections have been found to be associated with geographical variations in transmission stability, level of endemicity, and/or entomological inoculation rate [30-32,34,36,41], which are also influenced by local climate and climatic trends [30].

In terms of anaemia, worldwide prevalence among pre-school aged children was estimated by the WHO using country-specific data spanning from 1993–2005 [2]. Similar to the global distribution of malaria, anaemia prevalence appears to be highest in Africa (67.6%, 95% C.I. 64.3-71.0), followed closely by South-East Asia (65.5%, 95% C.I. 61.0-70.0) [2]. At the country level, inverse associations were found between the prevalence of moderate anaemia and the level of urbanization in two studies [21,42]. Using geographically weighted regression (GWR), Mainardi et al. also reported a significant association between access to a water source (defined by the median number of minutes to attain) and the prevalence of moderate anaemia (defined as haemoglobin 7–9.9 g/dl) among children < 5 years of age (p < 0.01), but not severe anaemia [21]. Two studies reported prevalence data for both malaria and anaemia [22,29]. Snow et al. compared the health outcomes of young children (0–4 years of age) from two East African communities with markedly different levels of malaria transmission– Kilifi, Kenya and Ifakara, Tanzania – and found that the site with a higher prevalence of severe anaemia also had higher parasitaemia [22]. Conversely, Greenwell et al. found the highest risk of severe anaemia among children in areas of moderate malaria prevalence [29].

## Discussion

### GIS applications and spatial analyses of malaria and anaemia among children

The literature reviewed herein has demonstrated that geo-spatial concepts and analysis techniques are generally used to describe geographical variation in an outcome measure, sometimes in concurrence with variations in other spatial and non-spatial factors such as time, or climate. Descriptive statistics were presented mainly through thematic maps (e.g. choropleth or dot density). Overall, the evidence has also indicated that geographic factors play a role in the prevalence of malaria and anaemia among young children living in low- and middle-income countries

Spatial analyses and statistical modeling have revealed both positive and negative associations between various geographic factors and the prevalence of malaria, or other related outcomes (such as malaria-related mortality). In some cases, the geographical factors that were associated with malaria prevalence (e.g. distance to larval habitat and transmission stability) were also modified by climatic trends such as seasonal rainfall and regional temperature gradients. Although GIS was not applied as extensively in the anaemia literature retrieved, there were notable similarities in the findings, namely the inverse association between anaemia prevalence and level of urbanization, as well as access to a community service such as piped water. In other words, anaemia has been found to be more common among rural and isolated communities with poorer access to a reliable source of water. The fact that anaemia and malaria share common biological pathways – through red blood cell turnover – and have similar continental-level spatial patterns (e.g. highest prevalence in both Africa and South-East Asia) suggests a geo-biological link that should be investigated further.

### Strengths and limitations

Approximately half of the peer-reviewed papers retrieved were published prior to the year 2000, and all studies were observational. The former may be a reflection of the search strategy used; however, we remain confident that the sources included, from both the peer-reviewed and grey literature, have provided a fair depiction of GIS applications in paediatric global health research pertaining to malaria and anaemia. In the latter case, the Cochrane Collaboration has tended to regard observational research as lower quality in terms of evidence for informing intervention effectiveness [43]. Considering that the investigation of geographical patterns and spatial relationships related to health outcomes lends itself almost exclusively to non-experimental study designs, using the Cochrane criteria for evaluating evidence may bias the results for a study towards ‘low’ quality when in fact it is closer ‘moderate’ or ‘high’ relative to other studies in the field, and after accounting for other factors that are more relevant to this topic area (rather than intervention research). GRADE and STROBE are published and verified approaches for evaluating research quality that were adapted in order to account for the potential bias mentioned above, as well as the variability in quality across studies, while maintaining transparency regarding the characteristics of the body of literature reviewed [16,17,43]. These novel assessment tools while innovative and potentially ground-breaking, in terms of their contribution to GIS literature reviews, should be further tested and formally evaluated for validity and reliability. Lastly, unlike the Cochrane method [43], quality ratings were not included as part of the relevance criteria. Without prior knowledge of the current range or nature of global paediatric health research that includes GIS, this methodological modification was considered important for determining the extent of research activity, as well as identifying potential opportunities to inform future research agendas in this area.

## Conclusions

Overall, we found that the investigation of geographic relationships with specific health outcomes has extended beyond simply mapping and describing spatial distribution patterns, to more complex analyses and predictive modelling to incorporate the effect of other environmental and spatial factors, such as regional variations in climate and distributions in population density. Bearing in mind the potential barriers of GIS use (e.g. lack of available data or prohibitive cost), it was somewhat discouraging that the application of GIS in paediatric anaemia and malaria research was so limited. Especially in the case of anaemia, as the burden of this disease among children under five years is equally if not even more widespread than malaria; and a plethora of prevalence and evaluation data exist that would be amenable to geographical mapping and spatial analysis. Current interest in the interaction between malaria and iron deficiency anaemia, may present an ideal opportunity to employ GIS to explore this relationship further. Such research could benefit local health officials and policy makers in malaria endemic areas who are still without definitive guidelines for planning and implementing anaemia control programs.

## Abbreviations

DHS: Demographic and health survey; ESRI: Environmental systems research institute; GIS: Geographical information systems; GRADE: Grades of recommendation, assessment, development and evaluation; GWR: Geographically weighted regression; MARA: Mapping malaria risk in Africa; MAP: Malaria atlas project; NCGIA: National centre for geographic information and analysis; STROBE: Strengthening the reporting of observational studies in epidemiology; UNICEF: United nations children’s fund; WHO: World health organization.

## Competing interests

Outside of the interests associated with their affiliations and the acknowledgements, the authors do not have financial or non-financial competing interests to disclose.

## Authors’ contributions

AMA conceived the project and developed the overall research plan and oversaw the study, conducted the research, synthesized and interpreted the data, and wrote the manuscript. NP conducted the research, and reviewed the manuscript. DCC was involved in project conception and study oversight, and contributed to various phases of the project from reviewing the protocol, conducting the research, and reviewing the manuscript. All authors read and approved the final manuscript.
